# Joint application of multiplex drop-off digital PCR, droplet digital PCR, and metagenomic next-generation sequencing for the diagnosis of suspected infectious diseases: A retrospective cohort study

**DOI:** 10.1016/j.jointm.2025.03.006

**Published:** 2025-05-14

**Authors:** Shanshan Jin, Shiyu Meng, Qiuping Huang, Hui Xie, Jingjing Zheng, Ruilan Wang

**Affiliations:** 1 Department of Critical Care Medicine, Shanghai General Hospital of Nanjing Medical University, Shanghai, China; 2 Department of Critical Care Medicine, Shanghai General Hospital, Shanghai Jiaotong University, School of Medicine, Shanghai, China

**Keywords:** Polymerase chain reaction, High-throughput nucleotide sequencing, Bacteremia, Etiology, Microbiological techniques

## Abstract

**Background:**

Critically ill patients in ICUs are highly vulnerable to infectious diseases. Early and accurate identification of pathogens is vital for initiating appropriate antimicrobial therapy. To evaluate the diagnostic effectiveness in patients with suspected infectious diseases; three different molecular technologies and conventional microbiological tests were used.

**Methods:**

A total of 97 individuals suspected of having infectious diseases were retrospectively enrolled from July 2023 to January 2024 at Shanghai General Hospital. Samples were collected for metagenomic next-generation sequencing (mNGS), droplet digital polymerase chain reaction (ddPCR), multiplex drop-off digital polymerase chain reaction (MDO-dPCR), and conventional microbiological tests (CMTs) for suspected pathogen detection. The diagnostic efficacies of the three molecular technologies and CMTs were compared, and the effects of their joint application on clinical outcomes were evaluated. Intergroup comparisons were performed using the Kruskal–Wallis test, with a *P*-value <0.05 considered statistically significant.

**Results:**

Joint detection exhibited a high negative predictive value. The sensitivity of MDO-dPCR, ddPCR, and mNGS was 52.6%, 48.5%, and 96.6%, respectively; and the corresponding specificity was 72.5%, 73.3%, and 50.0%. A positive correlation was observed between pathogen copies detected using MDO-dPCR and procalcitonin (Pearson’s *ρ*=0.21, *P*=0.039), acute physiology and chronic health evaluation II (Pearson’s *ρ*=0.24, *P* =0.018), and sequential organ failure assessment (Pearson’s *ρ*=0.25, *P*=0.012). Therapeutic regimens were adjusted in 51.5% of the patients (50/97) based on the results of the combination tests.

**Conclusions:**

In the present study, we highlighted the significance of molecular technologies for the early diagnosis of patients with suspected infections. These technologies can serve as a complement to CMTs and should be implemented promptly to guide clinicians in providing timely and effective anti-infective treatments. Future studies should aim to confirm these findings in large-scale clinical trials to refine diagnostic protocols, while also incorporating cost-utility analyses.

## Introduction

Infectious diseases persist as significant contributors to morbidity and mortality worldwide, particularly in intensive care units (ICUs).^[^^1^^,^^2^^]^ Bacteremia is a serious complication that can develop from localized infections, especially in patients with underlying conditions, such as diabetes mellitus, advanced tumors, or chronic kidney disease. Prompt diagnosis and accurate pathogen identification are essential for the effective use of antimicrobials, which helps shorten disease duration, improve patient outcomes, and limit the development of drug-resistant pathogens. The 2021 International Guidelines for the Management of Sepsis and Septic Shock emphasize the importance of administering antimicrobials as quickly as possible, ideally within the first hour of the diagnosis of sepsis or septic shock.^[^^3^^]^ Rapid initiation of antibiotic therapy is especially vital for at-risk adults because early treatment is key to improving patient outcomes. With the advancement and diversification of clinical testing methods, many techniques have become available. Conventional blood and non-blood cultures are considered the gold standard for identifying the causative pathogens in the diagnosis of suspected infections. However, conventional microbiological tests (CMTs) such as blood cultures (BCs) or other body fluid cultures often have a low detection rate for positive results and an extended turnaround time. This could be attributed to the low bacterial content and extensive use of antibiotics, which can adversely affect patient treatment and prognosis.^[^^4^^]^

Early detection of the pathogen in blood samples, while the infection is still confined to specific areas such as the lungs, abdomen, or urinary tract, seems to be a sensible strategy to ensure timely diagnosis and treatment of the suspected infectious diseases.^[^^5^^]^ Advancements in molecular technology have led to the development of new methods for rapid diagnosis of pathogens. These methods can detect trace amounts of pathogen DNA fragments even in cases where antibiotics have been used.^[^^6^^]^ Droplet digital polymerase chain reaction (ddPCR) and metagenomic next-generation sequencing (mNGS) are advanced molecular technologies that are commonly used to detect pathogens.^[^^7^^,^^8^^]^ Multiplex drop-off digital polymerase chain reaction (MDO-dPCR) assays have been used to detect various gene mutations in patients with colorectal cancer and have been increasingly applied in recent years for the detection of pathogens.^[^^9^^]^ mNGS has shown a significantly higher detection rate than traditional BCs and can be used to identify a broad spectrum of pathogens, including rare clinical bacteria and new strains.^[^^7^^]^ ddPCR works by distributing nucleotide-containing samples into thousands of independent partitions. This method is less affected by PCR inhibitors and is more responsive to microbial genes, leading to increased sensitivity and precision.^[^^8^^]^ MDO-dPCR has been used for pathogen detection because of its high sensitivity.^[^^9^^]^

In the present study, we performed a retrospective analysis to determine whether incorporating mNGS, ddPCR, and MDO-dPCR as diagnostic tools could help narrow the diagnostic gap for suspected infectious diseases.

## Methods

### Study design

This retrospective cohort study was conducted at a single center, without a control group, in the Department of Critical Care Medicine at Shanghai General Hospital from July 2023 to January 2024 in Shanghai, China. All the data used in this study were retrieved from an electronic medical records system. The study protocol was approved by the Institutional Review Board and Ethics Committee (No. 20230720065958542) of Shanghai General Hospital. Written informed consent for participation in the study was obtained from all patients or their legal representatives in accordance with ethical standards.

### Patients

Patients aged ≥18 years who were suspected of having infectious diseases were included in this retrospective study. Clinical suspicion of infection was based on clinical signs and symptoms, inflammatory markers, and the onset of organ dysfunction: (1) a peak body temperature surpassing 38.5 °C, (2) an increased white blood cell count, along with elevated C-reactive protein (CRP) and procalcitonin (PCT) levels, unexplained by noninfectious factors. Daily assessments of organ dysfunction and illness severity were conducted using the acute physiology and chronic health evaluation II (APACHE II) and sequential organ failure assessment (SOFA) scoring systems. The exclusion criteria were as follows: (1) patients with insufficient clinical information and (2) patients or their legal representatives who refused to participate.

### Sample collection time

Peripheral blood samples were collected on the day of enrollment for mNGS, ddPCR, and MDO-dPCR testing. On day 5 after enrollment, peripheral blood was collected again specifically for MDO-dPCR testing.

## Outcomes

The primary goal of this study was to evaluate the diagnostic accuracy of mNGS, ddPCR, and MDO-dPCR compared to that of CMTs. The secondary endpoint was the positive effect on the treatment outcomes.

### Pathogen detection using CMTs, mNGS, ddPCR, and mdo-dpcr

#### Routine culture and CMTs

All the enrolled patients underwent routine cultures to identify bacteria or fungi. Conventional microbiological methods, including blood/non-blood cultures and serological examinations, were performed as clinically indicated. For clinically suspected infection, whole blood samples and other body fluids (including bronchoalveolar lavage fluid [BAL], cerebrospinal fluid [CSF], abdominal fluid, liver abscess puncture fluid, bile, and urine) were subjected to CMTs. Blood and other bodily fluids were cultured in BC bottles. The BCs were incubated at 37 °C using the BacT/ALERT® 3D System (bioMérieux, Marcy-l'Étoile, France). Once a positive signal was detected, Gram staining was performed, and the sample was subcultured on a Columbia blood agar plate at 37 °C with 5% CO_2_. Pathogen identification was performed using matrix-assisted laser desorption/ionization time-of-flight mass spectrometry (UltrafleXtreme™ MALDI TOF/TOF; Bruker, Billerica, MA, USA) following overnight incubation.

#### Sample processing and DNA extraction of mNGS, ddPCR and mdo-dpcr

With the patient’s consent, blood samples were collected and analyzed using mNGS, ddPCR, and MDO-dPCR. If infections at other sites were suspected, specimens from these areas were also submitted for mNGS testing.

For mNGS, 3 mL BAL samples were collected from the patients following standard procedures. A horizontal vortex mixer was used to agitate a 1.5 mL microcentrifuge tube containing 0.6 mL of the sample, enzyme, and 1 g of 0.5 mm glass beads vigorously for 30 min at 2800–3200 rounds/min. After that, a 0.3 mL piece of the material was moved to a fresh 1.5 mL microcentrifuge tube, DNA was then extracted in accordance with the manufacturer's instructions using the TIANamp Micro DNA Kit (DP316; TIANGEN BIOTECH, Beijing, China). To separate the plasma, blood samples were collected in K2-ethylenediaminetetraacetic acid (EDTA) tubes and centrifuged at 1600 × *g* for 10 min. DNA was extracted from a 300 µL plasma sample using plant-derived DNA fragments as an internal standard. A nucleic acid extraction and purification kit (RM0184; BGI, Shenzhen, China) was used to extract DNA from the samples, positive controls, and negative controls in accordance with the manufacturer's instructions. A Qubit fluorometer (Thermo Fisher, Waltham, MA, USA) was utilized to assess the concentration of DNA. Samples that had a DNA concentration higher than 0.1 ng/µL were permitted to move on to the subsequent phase.

Peripheral blood samples were first centrifuged at 1600 × *g* for 15 min at 4 °C to prepare them for ddPCR analysis. Next, 2 mL of the supernatant was added to 10 mL of internal control and placed in an Auto-Pure 10 B nucleic acid purification system (Hangzhou Allsheng Instruments Co., Ltd., Hangzhou, China) according to the manufacturer's protocol. This allowed for the extraction of cell-free DNA (cfDNA) using a Magnetic Serum/Plasma DNA Kit (Beijing, China). After being eluted into 60 mL of 10 mg Tris-EDTA buffer—a solution that dissolves DNA and RNA while shielding it from degradation—the cfDNA was kept at −80 °C for subsequent examination. A 5-channel fluorescent ddPCR machine (Pilot Gene Technologies, Hangzhou, China) was used. Supplementary Table S1 lists the specifics of the target pathogen panels. GeneDPT software (Pilot Gene Technologies, Hangzhou, China) was used to analyze the results. The target pathogen detection threshold was set at 0.7 copies/mL in accordance with manufacturer specifications, and ddPCR results were deemed positive if the concentration surpassed this threshold.

For MDO-dPCR, using EDTA blood collection tubes, a 5 mL peripheral blood sample was taken (Pregene Biotechnology, Shenzhen, China) and processed within 2 h of collection. All subsequent steps followed the same protocol as that used for ddPCR. The number of droplets in each reaction tube should be maintained between 12,000 and 22,000 because deviations beyond this range could indicate issues with the detection process. In the negative control, each channel contained fewer than 5 positive droplets. If any channel in any tube contains 5 or more positive droplets, it suggests contamination with positive fragments during detection, rendering the experimental results invalid and requiring a repeat test. The target pathogen panels are listed in detail in Supplementary Table S2.

#### Sequencing and bioinformatic analysis of mNGS

Before the library building, all DNA samples, except those isolated from plasma samples, were subjected to fragmentation and purification. Sequencing libraries were constructed using the end-repair technique with a PMSeq kit (RM0438; BGI, Shenzhen, China) on the MGISP-100 platform (BGI, Shenzhen, China). Sequencing performed using an MGISEQ-2000 platform (BGI, Shenzhen, China). The sequencing data were processed automatically to generate the results. Human genome sequences were filtered using BWA alignment (https://bio-bwa.sourceforge.net/). After eliminating low-complexity sequences, the remaining sequences were compared to a specialized microorganism database to ascertain the quantity of aligned microbial sequences. The reporting team identified possible infections by analyzing the sequencing count and clinical background information.

### Statistical analysis

We adopted SPSS version 26.0 (IBM Corp., Armonk, NY, USA) and R version 4.3.2 (The R Foundation for Statistical Computing, Vienna, Austria) for our statistical analysis. The figures were produced using Prism 10.2.0 and R version 4.3.2. Continuous variables for general characteristics and laboratory tests were presented as mean±standard deviation if they met the Kolmogorov-Smirnov test for normality, and as median (interquartile range [IQR]) if they did not. The results were considered statistically significant with a *P*-value of <0.05. Intergroup differences were analyzed using the Kruskal–Wallis test.

## Results

### Characteristics of the patients

A total of 97 patients suspected of having an infectious disease were included, including 21 female patients (21.6%). The median age of the patients was (64.3±14.4) years. The three most common comorbidities were hypertension (48.5%), diabetes mellitus (30.9%), and active malignant disease (28.9%). The median plasma levels of CRP and PCT were 137.3 (IQR: 68.0–242.5) mg/L and 1.3 (IQR: 0.5–6.6) µg/L, respectively. The SOFA and APACHE II scores were (9.6±5.3) and (18.9 ± 9.2), respectively. Among 97 patients, the infection-related mortality rate at 28 days was 24.7%, whereas the overall mortality rate at 28 days was 29.9%. Additionally, 58 patients (59.8%) received vasopressors, 72 (74.2%) received intubation and invasive ventilation, and 19 (19.6%) received renal replacement therapy. Table 1 provides a summary of the patients' clinical and demographic features.

**Table 1:** Demographics and clinical characteristics of 97 patients in intensive care unit.

Clinical characteristics	Value (*n*=97)
Female	21 (21.6)
Age (years)	64.3±14.4
BMI (kg/m^2^)	23.0±5.8
Comorbidity	
Hypertension	47 (48.5)
Stroke	12 (12.4)
CVD	25 (25.8)
COPD	10 (10.3)
CKD	15 (15.5)
Diabetes mellitus	30 (30.9)
Liver cirrhosis	3 (3.1)
Solid organ transplantation	3 (3.1)
Hematopoietic stem cell transplantation	2 (2.1)
Active malignant disease	28 (28.9)
Physical examination findings	
Body temperature ( °C)	38.6±1.0
Heart rate (beats/min)	109.5±18.4
Mean arterial pressure(mmHg)	86.3±20.5
Blood laboratory examination	
White blood cell count (× 10^9^/L)	11.1 (6.7–18.8)
Absolute neutrophil count (× 10^9^/L)	9.8 (5.6–16.9)
Absolute lymphocyte count (× 10^9^/L)	0.6 (0.4–0.9)
Hemoglobin (g/L)	103.0 (79.0–119.0)
Platelet count (× 10^9^/L)	120.0 (70.0–185.5)
Serum creatinine (µmol/L)	106.4 (74.0–175.2)
Total bilirubin (µmol/L)	19.4 (12.5–33.5)
Serum lactate (mmol/L)	1.5 (0.9–3.2)
C reactive protein (mg/L)	137.3 (68.0–242.5)
Procalcitonin (µg/L)	1.3 (0.5–6.6)
Clinical metrics	
Vasopressors	58 (59.8)
Intubation and invasive ventilation	72 (74.2)
Renal replacement therapy	19 (19.6)
SOFA score	9.6±5.3
APACHE II core	18.9±9.2
28-day infection-related mortality	24 (24.7)
Total 28-day mortality	29 (29.9)
Hospitalization costs (CNY × 10^4^)	16.3±13.0

Data are presented as *n*(%), mean±standard deviation, and median (interquartile range).

APACHE II: Acute physiology and chronic health evaluation II; BMI: Body mass index; COPD: Chronic obstructive pulmonary disease; CKD: Chronic kidney disease; CNY: Chinese Yuan; CVD: Cardiovascular disease; DM: Diabetes mellitus; SOFA: Sequential organ failure assessment.

**Figure 1:**
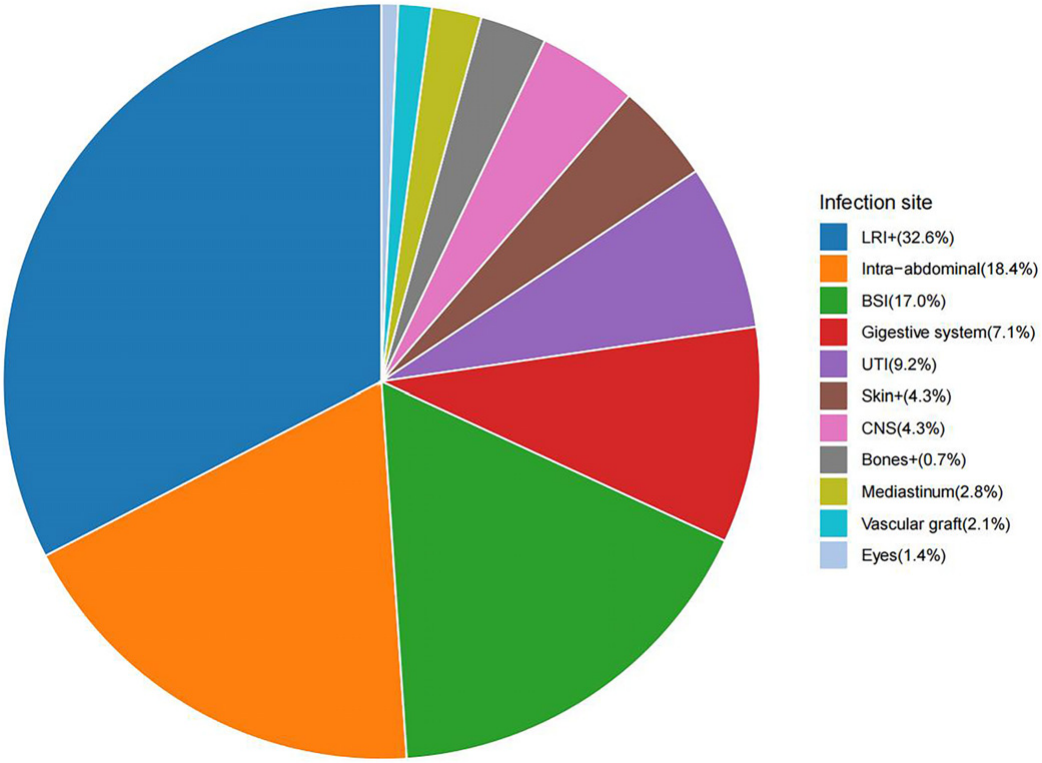
The infection site of 97 patients. Bones+: Infections of bones, joints, and related organs; BSI: Bloodstream infections; CNS: Meningitis and other bacterial CNS infections; Intra-abdominal: Peritoneal and intra-abdominal infections; LRI+: Lower respiratory infections and all related infections in the thorax; Skin+: Bacterial infections of the skin and subcutaneous systems; UTI: Urinary tract infections and pyelonephritis.

Blood, sputum, nasopharyngeal swabs, puncture fluids, bronchoalveolar lavage fluid, aspirates, and other clinical specimens were collected for testing according to the suspected type of infection. The infection site categories include infections of bones and joints (0.7%), meningitis and other bacterial central nervous system (4.3%), the skin (4.3%), urinary tract (9.2%), bloodstream (17.0%), intra-abdominal (18.4%), lower respiratory (32.6%), eyes (1.4%), vascular graft (2.1%), mediastinum (2.8%) and digestive system (7.1%). The pie chart provides a proportional representation of these infection sites, with each segment corresponding to the percentage of patients affected by each infection type. (Figure 1).

### Pathogens identified by CMTs, confirmed by mNGS, ddPCR, and Mdo-dPCR

All the 97 patients underwent MDO-dPCR. Among them, 48 patients were also tested using ddPCR, and 35 patients were tested using mNGS. Table 2 lists 22 positive samples from 17 patients in whom pathogenic microorganisms were identified using CMTs. Among the 22 samples, there were 7 blood, 7 BAL, 3 abdominal fluid, 2 bile, 1 urine, 1 liver abscess fluid, and 1 CSF samples. Gram-negative bacteria included *Escherichia coli, Acinetobacter baumannii, Klebsiella pneumoniae*, and *Aeromonas hydrophila*. The Gram-positive bacterial strains identified were *Enterococcus faecalis* and *Staphylococcus aureus*. Fungal strains comprised *Candida albicans, Candida glabrata*, and *Talaromyces marneffei*. Fourteen samples were concordantly positive with MDO-dPCR, eleven samples were concordantly positive with ddPCR, and thirteen samples were concordantly positive with mNGS.

**Table 2:** Pathogen detection among MDO-dPCR, ddPCR and mNGS in patients with positive culture.

Patient ID	Specimens	Culture results	MDO-dPCR	ddPCR	mNGS (Blood/BAL)
2	Blood	*Escherichia coli*	*Escherichia coli*	*Escherichia coli*	*–*
4	Abdominal puncture liquid	*Escherichia coli; Enterococcus faecalis*	*Escherichia coli; Enterococcus faecalis*	*–*	*–*
5	Bile	*Klebsiella pneumoniae*	*Klebsiella pneumoniae*	*Klebsiella pneumoniae*	*–*
7	Bile/ Abdominal puncture liquid	*Klebsiella pneumoniae*	*Klebsiella pneumoniae*	*Klebsiella pneumoniae*	*–*
9	BAL	*Acinetobacter baumannii; Klebsiella pneumoniae*	*Acinetobacter baumannii;*	*Acinetobacter baumannii;*	*Acinetobacter baumannii; Klebsiella pneumoniae*
32	Blood	*Escherichia coli*	*Escherichia coli*	*Escherichia coli*	*Escherichia coli; Bacillus cereus*
33	BAL/urine	*Klebsiella pneumoniae*	*Klebsiella pneumoniae*	*–*	*Klebsiella pneumoniae; Candida albicans*
41	Blood/Abdominal puncture liquid	*Aeromonas hydrophila*	*Enterococcus faecalis*	*–*	*Aeromonas hydrophila; Enterococcus faecalis, CMV*
53	BAL	*Klebsiella pneumoniae*	*Klebsiella pneumoniae*	*Klebsiella pneumoniae*	*Klebsiella pneumoniae*
54	Liver puncture liquid	*Klebsiella pneumoniae; Escherichia coli*	*Klebsiella pneumoniae: Escherichia coli*	*Klebsiella pneumoniae: Escherichia coli*	*Klebsiella pneumoniae: Escherichia coli; Bacteroides fragilis; Prevotella*
55	Blood	*Klebsiella pneumoniae*	*Klebsiella pneumoniae*	*Klebsiella pneumoniae*	*Klebsiella pneumoniae; Clostridium perfringens*
75	Blood	*Talaromyces marneffei*	*–*	*–*	*Talaromyces marneffei*
79	Blood/ BAL	*Candida glabrata; Candida albicans*	*Candida glabrata*	*Candida glabrata; Candida albicans; Klebsiella pneumoniae*	*Candida glabrata; Candida albicans; Mycobacterium kansasii*
80	CSF	*Klebsiella pneumoniae*	*Klebsiella pneumoniae*	*Klebsiella pneumoniae*	*Klebsiella pneumoniae*
81	BAL	*Staphylococcus aureus*	*Staphylococcus aureus*	*Staphylococcus aureus*	*Staphylococcus aureus; Hemophilus influenzae*
86	Blood/ BAL	*Staphylococcus aureus*	*Escherichia coli; Hemophilus influenzae*	*–*	*Staphylococcus aureus; IAV*
92	BAL	*Staphylococcus aureus; Candida albicans*	*Staphylococcus aureus*	*–*	*Staphylococcus aureus; Candida albicans; Enterococcus faecium; EBV; IAV*

BAL: Bronchoalveolar lavage fluid; CMV: Cytomegalovirus; CSF: Cerebrospinal fluid; ddPCR: Droplet digital polymerase chain reaction; EBV: Epstein–Barr virus; IAV: Influenza A virus; MDO-dPCR: Multiplex drop-off digital polymerase chain reaction; mNGS: Metagenomic next-generation sequencing.

### Pathogens detected by three molecular tests

**Figure 2:**
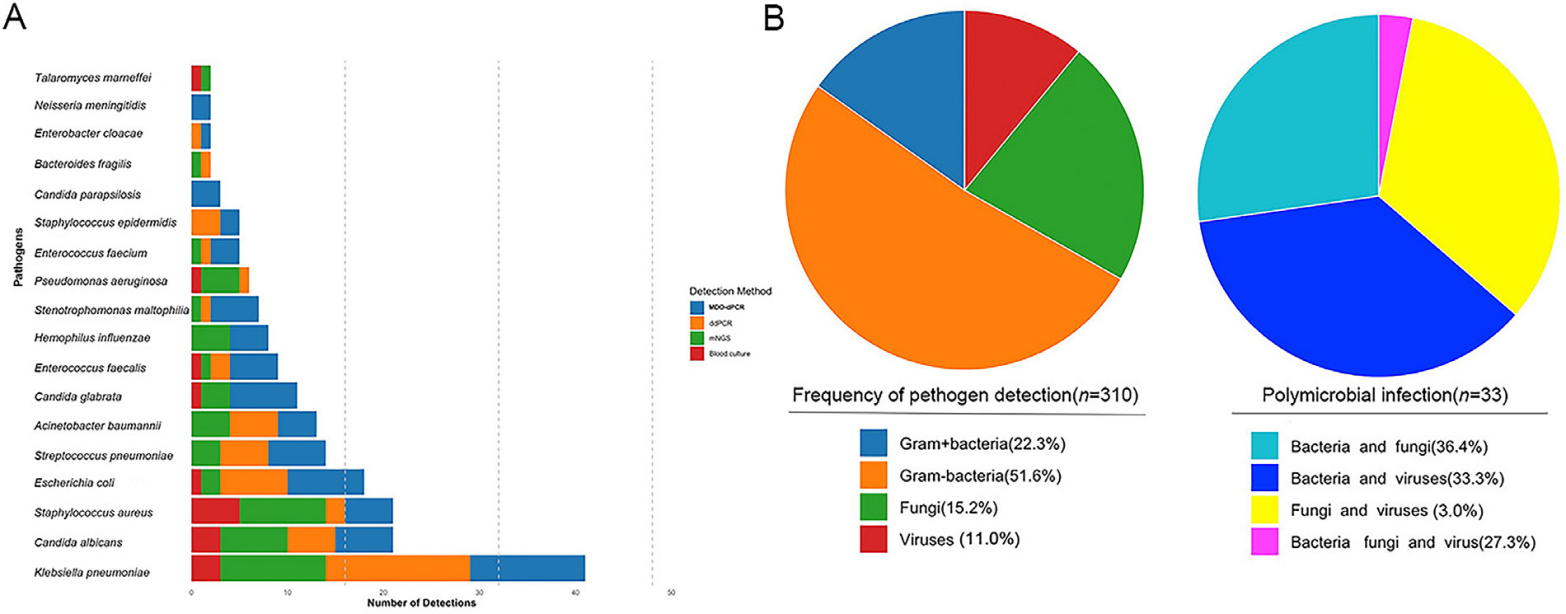
Pathogens detected by MDO-dPCR, ddPCR, and mNGS. ddPCR: Droplet digital polymerase chain reaction; MDO-dPCR: Multiplex drop-off digital polymerase chain reaction; mNGS: Metagenomic next-generation sequencing.

MDO-dPCR was used to identify 15 pathogens. The most common Gram-negative bacterial strains were *Klebsiella pneumoniae* (*n*=12), *Escherichia coli* (*n*=8), and *Stenotrophomonas maltophilia* (*n*=5). Among the 21 Gram-positive bacterial strains, the leading pathogens were *Streptococcus pneumoniae* (*n*=6), *Enterococcus faecalis* (*n*=5), and *Staphylococcus aureus* (*n*=5). The fungal infections detected were *Candida albicans* (*n*=6), *Candida glabrata* (*n*=7), and *Candida parapsilosis* (*n*=3) (Figure 2).

The top three Gram-negative bacteria detected by ddPCR were *Klebsiella pneumoniae* (*n*=15), *Escherichia coli* (*n*=7), and *Acinetobacter baumannii* (*n*=5). The top three Gram-positive bacteria were *Streptococcus pneumoniae* (*n*=5), *Staphylococcus epidermidis* (*n*=3), and *Staphylococcus aureus* (*n*=2). The top one fungal pathogen was *Candida albicans* (Figure 2).

**Figure 3:**
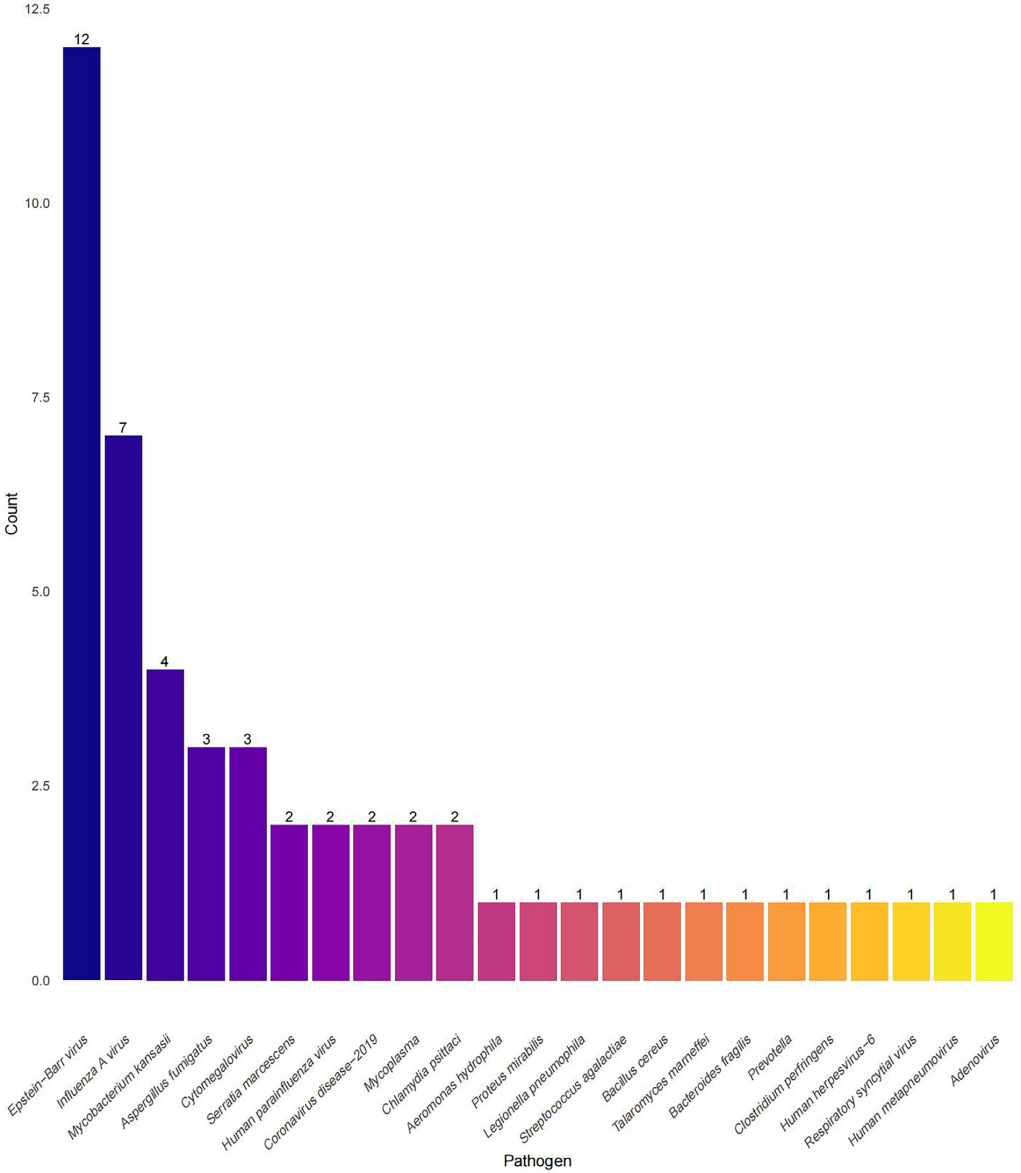
Pathogen detected by mNGS (*n*=52).

Using mNGS, 36 pathogens in all were identified using mNGS. *Klebsiella pneumoniae* (*n* =11), *Pseudomonas aeruginosa* (*n*=4), *Acinetobacter baumannii* (*n*=4), *Haemophilus influenzae* (*n*=4), and *Mycobacterium kansasii* (*n*=4) were the most common. Gram-negative bacteria. *Staphylococcus aureus* (*n*=9) and *Streptococcus pneumoniae* (*n*=3) were the predominant Gram-positive bacteria. Fungal pathogens identified by mNGS included *Candida albicans* (*n*=7), *Candida glabrata* (*n*=3), *Aspergillus fumigatus* (*n*=3), and *Talaromyces marneffei* (*n*=1). mNGS demonstrated superior detection power for specific pathogens including *Talaromyces marneffei*, Epstein–Barr virus, cytomegalovirus, human herpesvirus-6, human parainfluenza virus, coronavirus disease 2019, influenza A virus, respiratory syncytial virus, human metapneumovirus, adenovirus, Mycoplasma, and *Chlamydia psittaci* (Figure 3).

**Figure 4:**
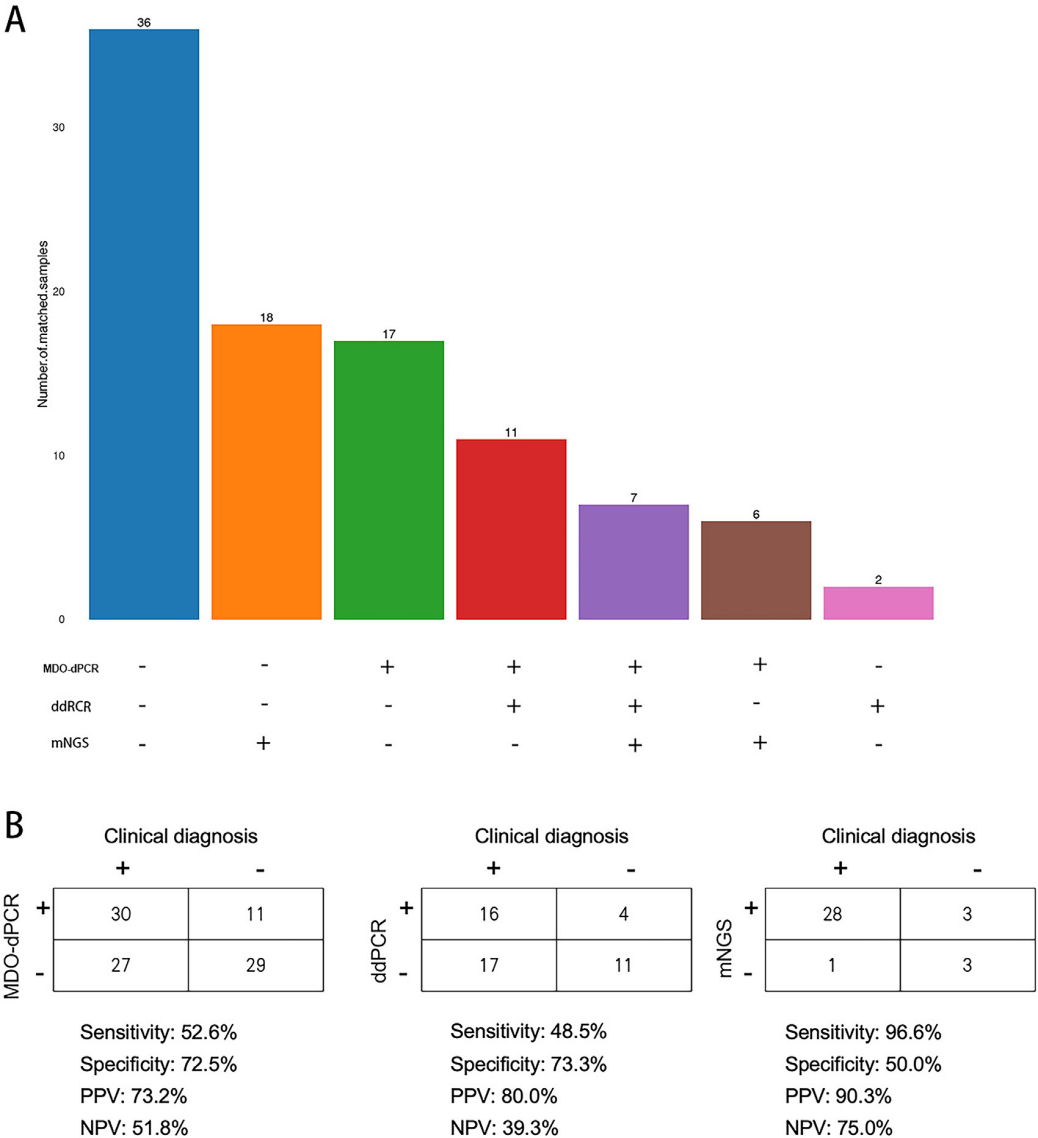
Diagnostic performance comparison among MDO-dPCR, ddPCR, and mNGS in suspected infection. A: Comparison of MDO-dPCR, ddPCR and mNGS results for suspected infection. B: Contingency tables for the clinical diagnosis with MDO-dPCR, ddPCR and mNGS. ddPCR: Droplet digital polymerase chain reaction; MDO-dPCR: Multiplex drop-off digital polymerase chain reaction; mNGS: Metagenomic next-generation sequencing; NPV: Negative predictive value; PPV: Positive predictive value.

Of the 97 patients, 61 developed infectious diseases, whereas 36 had noninfectious diseases. Seven patients had triple-positive results, six patients had positive findings from both mNGS and MDO-dPCR, 11 patients had positive findings from both MDO-dPCR and ddPCR, 18 patients had positive results from only mNGS, 17 patients had positive results from only MDO-dPCR, and two patients had positive results from only ddPCR. The MDO-dPCR assay had a sensitivity, specificity, predictive value (PPV), and negative predictive value (NPV) of 52.6%, 72.5%, 73.2%, and 51.8%, respectively. ddPCR had a sensitivity, specificity, PPV, and NPV of 48.5%, 73.3%, 80.0%, and 39.3%, respectively. mNGS had sensitivity, specificity, PPV, and NPV of 96.6%(the highest), 50.0%, 90.3% and 75.0%, respectively (Figure 4).

In the sex-based analysis, the sensitivity of MDO-dPCR was higher in males (54.6%) than in females (46.2%), whereas the specificity remained similar between the two groups. A similar trend was observed for ddPCR, where males exhibited lower sensitivity (46.2%) than females (57.1%), whereas the specificity was nearly identical. mNGS demonstrated the highest sensitivity among all the methods, with males (90.5%) performing slightly better than females (88.9%) (Supplementary Table S3).

Age-based subgroup analysis revealed differences in diagnostic accuracy (Supplementary Table S4). For MDO-dPCR, sensitivity was lower in patients aged <64 years (38.9%) than in those aged ≥ 64 years (59.0%). Similarly, ddPCR exhibited higher sensitivity in the ≥64 years group (66.7%) than in the <64 years group (38.1%). For mNGS, the sensitivity remained consistently high across both age groups.

### Correlation analysis between MDO-dPCR and laboratory inflammatory markers

**Figure 5:**
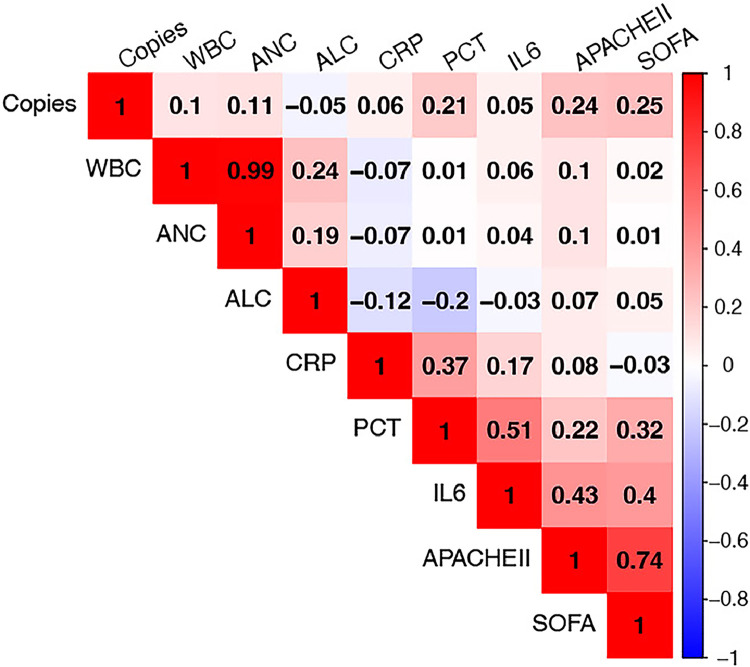
Heatmap of Pearson correlation coefficients among pathogen copy number and clinical indicators, including inflammatory markers and severity scores. The vertical and horizontal axes both represent the same set of variables (Copies, WBC, ANC, ALC, CRP, PCT, IL-6, APACHE II, SOFA), and each cell represents the correlation between the corresponding pair. Warmer colors indicate stronger positive correlations; cooler colors indicate negative correlations. ANC: Absolute neutrophil count; ALC: Absolute lymphocyte count; APACHE II: Acute physiology and chronic health evaluation II; CRP: C-reactive protein; IL: Interleukin; PCT: Procalcitonin; SOFA: Sequential organ failure assessment; WBC: White blood cell.

The correlation between pathogen copies detected by MDO-dPCR in the blood and clinical indicators (white blood cell [WBC], absolute lymphocyte count [ALC], absolute neutrophil count [ANC], CRP, PCT, interleukin-6 [IL-6], APACHE II, and SOFA) was calculated. Our analysis revealed positive correlations between pathogen copy number and PCT (Pearson’s *ρ*=0.21, *P*=0.039), APACHE II (Pearson’s *ρ*=0.24, *P*=0.018), and SOFA scores (Pearson’s *ρ*=0.25, *P*=0.012). Conversely, the correlations between pathogen load and WBC (Pearson’s *ρ*=0.10, *P*=0.330), ANC (Pearson’s *ρ*=0.11, *P*=0.272), ALC (Pearson’s *ρ*=−0.05, *P*=0.634), CRP (Pearson’s *ρ*=0.06, *P*=0.562), and IL-6 (Pearson’s *ρ*=0.05, *P*=0.464) levels were weak and not statistically significant (Figure 5).

**Figure 6:**
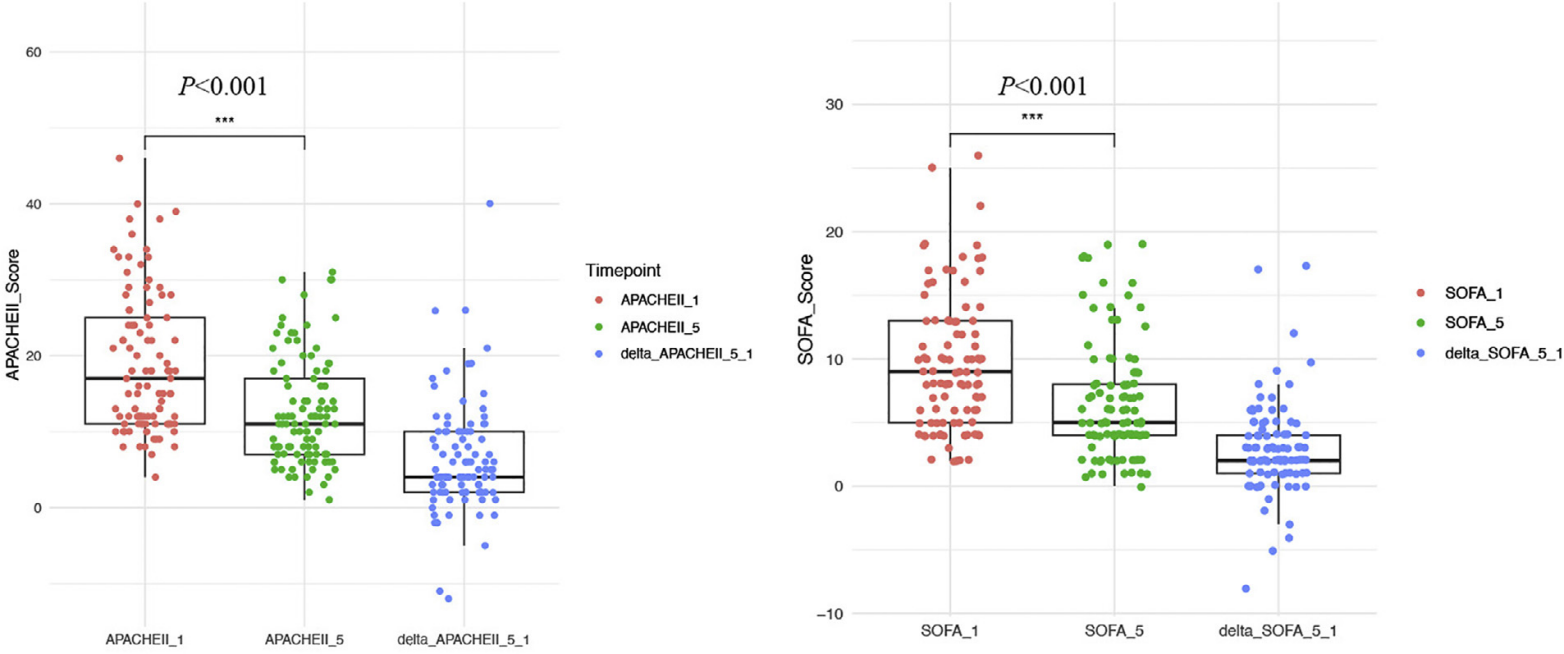
Changes in APACHE II and SOFA scores over the treatment period. APACHE: Acute physiology and chronic health evaluation; SOFA: Sequential organ failure assessment.

During the treatment period, we observed reductions in SOFA and APACHE II scores (Figure 6). We observed significant changes in both the APACHE II and SOFA scores over time. The APACHE II score on day 1 (APACHE_II_1) was significantly higher than that on day 5 (APACHE_II_5), with a marked reduction in scores observed between these two timepoints (*P* <0.001). The △APACHEII_5_1 values, representing the change in APACHE II scores from day 1 to day 5, emphasize the improvement in patients' conditions. The SOFA score showed a reduction from day 1 (SOFA_1) to day 5 (SOFA_5), again with a significant difference (*P* <0.001). The △SOFA_5_1, reflecting the change in SOFA scores from day 1 to day 5, showed a trend toward clinical improvement over the treatment period.

**Figure 7:**
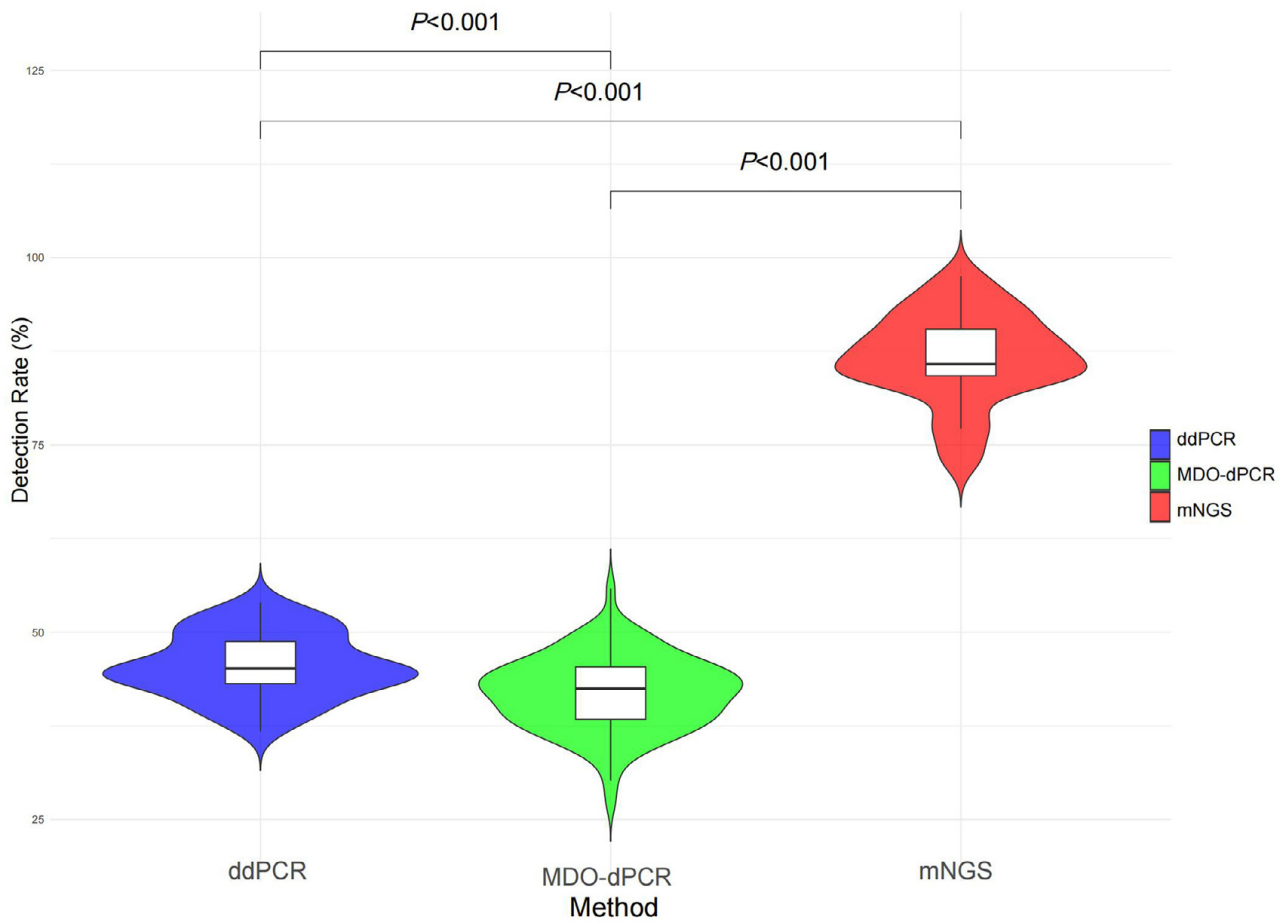
Violin plot comparing the detection rates of mNGS, MDO-dPCR, and ddPCR. ddPCR: Droplet digital PCR; MDO-dPCR: Multiplex drop-off digital PCR; mNGS: Metagenomic next-generation sequencing.

Regarding the detection rates of the three diagnostic methods, mNGS achieved the highest detection rate at 86.1%, significantly outperforming MDO-dPCR (42.3%) and ddPCR (45.5%). There was a statistically significant difference between the three groups (*P* <0.001). Subsequent pairwise comparisons confirmed that mNGS exhibited significantly higher detection rates than MDO-dPCR (*P* <0.001) and ddPCR (*P* <0.001). MDO-dPCR showed a slight but statistically significant difference compared to ddPCR (*P* <0.001) (Figure 7).

### Quantitative copy number changes in MDO-dPCR during antibiotic treatment

**Figure 8:**
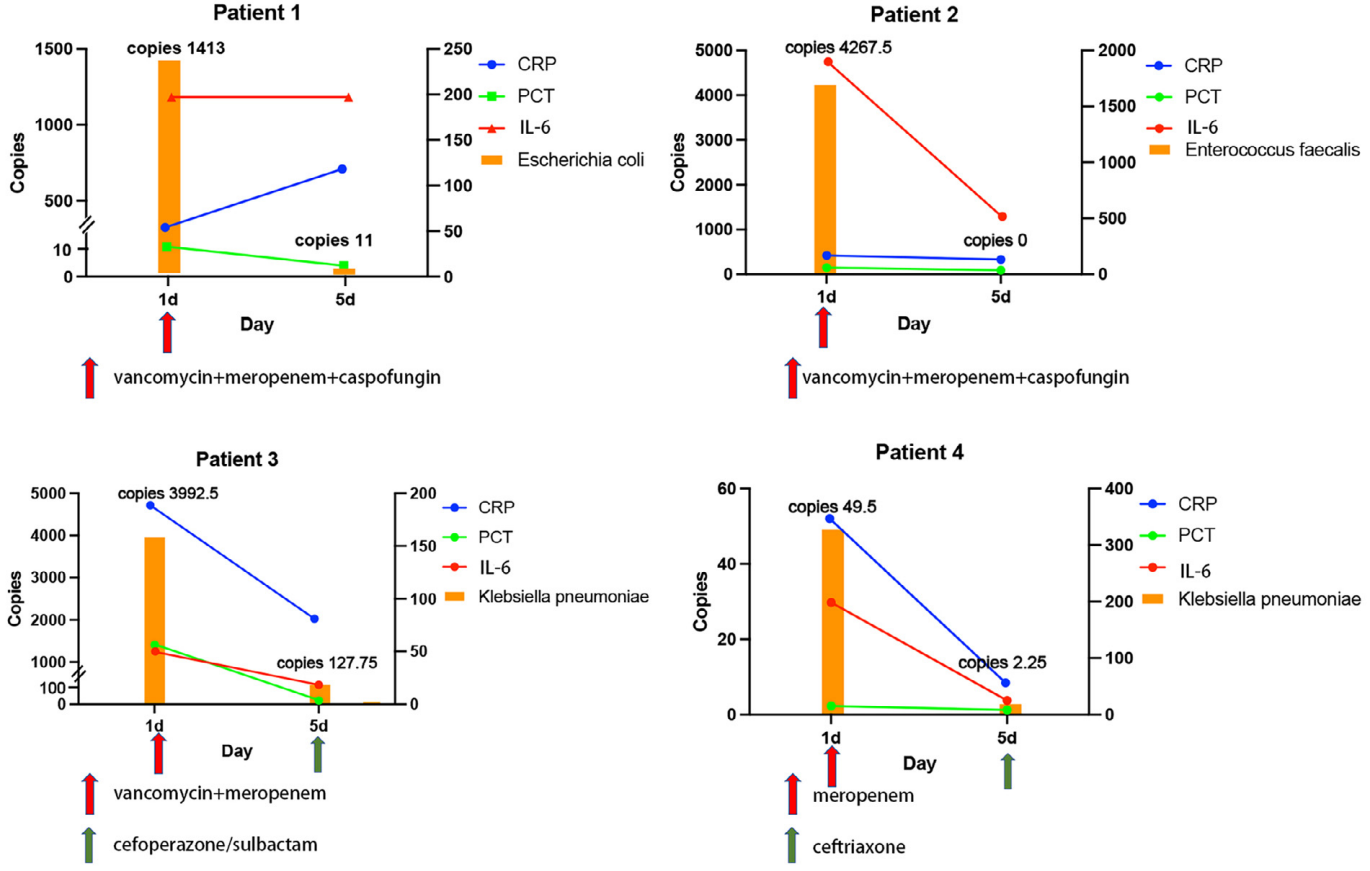
Temporal dynamics of pathogen load (left Y-axis, orange bars; expressed as copies) and inflammatory biomarkers (right Y-axis) in four patients from Day 1 to Day 5 following antimicrobial treatment. The right Y-axis represents serum levels of C-reactive protein (CRP, mg/L; blue), procalcitonin (PCT, ng/mL; green), and interleukin-6 (IL-6, pg/mL; red). Antimicrobial regimens are indicated with arrows. Pathogen species and their initial and final copy numbers are annotated above and below each bar. CRP: C-reactive protein; IL: Interleukin; PCT: Procalcitonin.

Moreover, we examined the relationship between bacterial loads ascertained through MDO-dPCR and indicators of inflammation over 5 days. In patient 1, who was treated with vancomycin, meropenem, and caspofungin, a notable decline in *Escherichia coli* copy numbers and inflammatory markers was observed. Patient 2, treated with vancomycin, meropenem, and caspofungin, showed a similar decline in *Enterococcus faecalis* copy numbers and inflammatory markers levers. Patient 3 was initially treated with vancomycin and meropenem. As *Klebsiella pneumoniae* copies and inflammatory markers declined, therapy was switched to cefoperazone/sulbactam. Finally, patient 4, treated with meropenem, demonstrates a decrease in *Klebsiella pneumoniae* copies and inflammatory markers. When patients received effective antimicrobial treatment, the MDO-dPCR copy numbers declined, which was consistent with the simultaneous decrease in CRP, PCT, and IL-6 values (Figure 8).

### Impact on antibiotic treatment

Among the 97 patients, based on the mNGS, ddPCR, and MDO-dPCR results, adjustments to the initial empirical antimicrobial agents were as follows: unnecessary agents were removed in 9 (9.3%) of cases, new agents were added in 4 (4.1%), and the agents were completely changed in 37 (38.1%). Additionally, there was no change in 47 (48.5%) of the cases. Table 3 summarizes the effects of mNGS, ddPCR, and MDO-dPCR on antimicrobial therapy for suspected infections.

**Table 3:** Impact of mNGS, ddPCR, and MDO-dPCR on antimicrobial treatment on suspected infections.

Modifications	mNGS (*n*=35)	ddPCR (*n*=48)	MDO-dPCR (*n*=97)
Remove unnecessary agents	4 (11.4)	2 (4.2)	3 (3.1)
Add agents	3 (8.6)	1 (2.1)	0
Change completely	11 (31.4)	9 (18.8)	17 (17.5)
No change	9 (25.7)	15 (31.3)	23 (23.7)

Data are presented as *n*(%).

ddPCR: Droplet digital polymerase chain reaction; MDO-dPCR: Multiplex drop-off digital polymerase chain reaction; mNGS: Metagenomic next-generation sequencing.

## Discussion

Advanced molecular technologies have the potential to significantly resolve diagnostic challenges associated with low pathogen detection rates, particularly in cases where traditional approaches are insufficient, such as culture-negative sepsis or polymicrobial infections, are insufficient. In this retrospective study, we compared the diagnostic efficacy of CMTs alone and in combination with mNGS, ddPCR, MDO-dPCR, and CMTs for a variety of infectious diseases. The detection rates were significantly higher for mNGS at 86.1% (including viruses), 45.5% for ddPCR, and 42.3% for MDO-dPCR, compared to 30.9% for CMTs. When the results from the CMTs were negative and the other three tests also yielded negative results, it suggested a high likelihood that the patient did not have an infectious disease. Among the 97 patients, 36 were diagnosed with noninfectious conditions. Thus, combined testing may have a high NPV. mNGS, ddPCR, and MDO-dPCR may be more sensitive than CMTs for diagnosing infectious diseases. These methods are less influenced by the survival status of pathogens and the history of antimicrobial treatment. This discovery underscores the ability of mNGS, MDO-dPCR, and ddPCR to identify pathogens regardless of the etiological classification, infection site, or source (community-acquired or nosocomial).

Our findings are consistent with those of several earlier studies comparing the differences between nucleic acid- and culture-based approaches for pathogen identification from whole blood.^[^^10^^,^^11^^]^ In a cohort study involving 150 patients with severe illnesses and 438 occurrences, the concordance rate between ddPCR and BCs was 63.9% (280/438). Targeted bacteria were identified in 40 patients (9.1%) using BCs and 180 patients (41.1%) using ddPCR.^[^^10^^]^ The sensitivity of ddPCR ranged from 58.8% to 86.7% compared to that of BCs, with an average of 72.5%. The specificity varied from 73.5% to 92.2%, with an average of 63.1%. In our study, ddPCR demonstrated a sensitivity, specificity, PPV, and NPV 48.5%, 73.3%, 80.0%, and 39.3%, respectively. The small sample size used in this study may be the reason for the lower results observed in comparison to earlier studies. A multicenter randomized controlled trial involving 349 patients with severe community-acquired pneumonia (SCAP) showed pathogen detection rates of 79.9% and 38.8% for the mNGS and CMTs groups, respectively (*P* <0.001).^[^^11^^]^ In terms of the types of pathogens detected, Gram-negative bacilli predominated, which is consistent with the composition of pathogenic microorganisms in ICU infectious diseases.^[^^12^^]^ MDO-dPCR and ddPCR demonstrated high detection rates for bacteria within their testing ranges, whereas mNGS exhibited a high detection rate for rare and uncultivable microorganisms. Our subgroup analysis showed that the diagnostic performance of MDO-dPCR, ddPCR, and mNGS varied according to age and sex, highlighting potential demographic influences. Sensitivity was generally higher in older patients (≥64 years) for both MDO-dPCR and ddPCR, whereas mNGS maintained consistently high sensitivity across all subgroups. These findings suggest that patient characteristics should be considered when interpreting diagnostic test results to ensure their applicability across diverse populations.

Infections induced by Gram-negative bacteria can result in serious and sometimes fatal illnesses. A previous study reported that Gram-negative bacteria were identified in 67% of patients, Gram-positive bacteria in 37%, and fungi in 16%.^[^^12^^]^ In the present study, a large proportion of the patients tested positive for Gram-negative bacterial infections, with *Klebsiella pneumoniae* having the highest detection rate. Previous analyses have demonstrated that the mortality rate associated with *Klebsiella pneumoniae* infections is high, and many survivors experience long-term complications. The mortality rate of patients with alcohol use disorder and diabetes can attain 100%.^[^^13^^]^ *Klebsiella pneumoniae* infections are associated with poor outcomes if the spread of the infection is not controlled within 48 h.^[^^14^^]^ Timely diagnosis of pathogens can save the lives of many patients and offer them a chance to recover.

PCR-based assays have demonstrated the capability of quickly detecting pathogens in whole blood samples.^[^^15^^,^^16^^]^ PCR-based bacterial load assays serve as a practical biomarkers for stratifying patients at a higher risk of complications and poor outcomes.^[^^17^^]^ In recent years, ddPCR has shown higher detection rates and faster results than traditional BCs, delivering accurate pathogen load data in approximately 2.5–3.0 h.^[^^10^^]^ It not only offers quantitative and precise data on the causative pathogen burden but also provides insight into the severity of inflammation and infection. ddPCR has the potential to provide clinicians with an early warning and monitor a patient's condition.^[^^18^^]^ The MDO-dPCR assay, another type of PCR-based test, exhibits high sensitivity and comprehensive mutation coverage for diagnosing colorectal tumors, particularly for detecting hotspot mutations.^[^^9^^]^ Using three-dimensional primers and a unique reaction system, this method effectively detects various pathogens, delivering results within 4 h with a minimum detection limit of 20 copies/mL. In a single cycle, mNGS technology can detect all DNA or RNA sequences in a sample in an unbiased manner. mNGS is a promising technique owing to its high sensitivity and quick turnaround time, making it valuable for early pathogen identification and for guiding appropriate antimicrobial therapy.^[^^19^^]^

An increase in pathogen load detected by MDO-dPCR correlated with higher inflammatory marker levels and SOFA and APACHE II scores. The bacterial load quantified by MDO-dPCR aligned with the trends in inflammatory indicators and was positively correlated with PCT levels. These findings suggest that MDO-dPCR can facilitate dynamic monitoring of pathogen clearance post-treatment, aid in disease progression monitoring, and optimize treatment strategies. In four patients where in whom pathogens were eventually identified through CMT culture, a reduction in inflammatory markers was observed following targeted therapy, which corresponded to a decrease in pathogen load. These findings are consistent with those of previous studies.^[^^6^^,^^20^^]^ The reduction in patients' SOFA and APACHE II scores may also indicate an improvement in their condition (Figure 6).

Antimicrobial therapy was modified in 50 of the 97 patients (51.2%) when we assessed the possible effect of joint testing on antimicrobial therapy for suspected infectious illnesses. In 9 patients (9.3%), unnecessary agents were discontinued; in 4 patients (4.1%), targeted agents were added and in 37 patients (38.1%), adjustments were made based on microbiological findings, which aligns with the results reported in previous studies.^[^^21^^]^ By utilizing three molecular diagnostic methods in combination with CMTs for mutual complement and cross-validation, we can implement an antimicrobial treatment plan based on the earliest results and confirm it with other methods. Consequently, our study effectively ruled out noninfectious patients, whereas for those suspected of infection, multiple methods were used to validate the findings.

This study has several limitations. First, it was important that this research is a retrospective study conducted at a single center with a limited sample size. The small sample size may have affected the consistency of microbial detection across the three molecular diagnostic techniques. The unintended predominance of older male patients (78.7%) may have limited the generalizability of our findings. Second, although our findings suggest that combined testing can identify mixed etiological infections in suspected infectious diseases, this study focused exclusively on common pathogen types in these mixed infections and did not specifically analyze the composition of the mixed etiologies. This limitation restricts our understanding of microbial interactions and their pathogenic significance. Third, this study lacks a parallel control group, which limits its ability to evaluate the true clinical impact of these diagnostic approaches on patient outcomes. A prospective cohort study with a larger sample size and a cost-effectiveness analysis is essential to validate the clinical utility of these molecular tests. Finally, although this study highlights the potential of combining three molecular diagnostic techniques, the economic feasibility of performing all three tests per patient remains a significant challenge. The high costs associated with these techniques may limit their widespread clinical application. Although these diagnostic methods have improved the detection of potentially infectious diseases, further research is required to comprehensively assess the cost-effectiveness of rapid molecular diagnostic testing.

## CRediT authorship contribution statement

**Shanshan Jin:** Writing – original draft, Visualization, Formal analysis, Data curation. **Shiyu Meng:** Software, Data curation. **Qiuping Huang:** Software, Methodology, Investigation. **Hui Xie:** Formal analysis, Data curation, Conceptualization. **Jingjing Zheng:** Writing – review & editing, Conceptualization. **Ruilan Wang:** Writing – review & editing, Supervision, Funding acquisition, Conceptualization.

## Appendix Supplementary materials

Image, application 1
