# Supplementary material for: Joint application of multiplex drop-off digital PCR, droplet digital PCR, and metagenomic next-generation sequencing for the diagnosis of suspected infectious diseases: A retrospective cohort study
Source: J Intensive Med. 2025 May 14;5(4):407–18. doi: 10.1016/j.jointm.2025.03.006 (PMC12572856; doi:10.1016/j.jointm.2025.03.006)
Supplement: Supplementary file 1 [file mmc1.docx]

Table S1. Pathogens Detection panels of ddPCR

| Target pathogens |
| --- |
| *Pseudomonas aeruginosa*  *Escherichia coli*  *Klebsiella pneumoniae*  *Acinetobacter baumannii*  *Staphylococcus aureus*  *Candida species*  *Enterococcus*  *Stenotrophomonas maltophilia*  *Enterobacter cloacae*  *Staphylococcus epidermidis*  *Proteus mirabilis*  *Proteus mirabilis*  *Bacteroides fragilis* |

Table S2. Pathogens Detection panels of MDO-dPCR

| Target pathogens |
| --- |
| *Staphylococcus aureus*  *Staphylococcus epidermidis*  *Hemophilus influenzae*  *Enterococcus faecium*  *Enterococcus faecalis*  *Stenotrophomonas maltophilia*  *Streptococcus pneumoniae*  *Acinetobacter baumannii*  *Klebsiella pneumoniae*  *Enterobacter cloacae*  *Escherichia coli*  *Pseudomonas aeruginosa*  *Serratia marcescens*  *Neisseria meningitidis*  *Candida albicans*  *Candida glabrata*  *Candida parapsilosis* |

Table S3. Diagnostic Performance of MDO-dPCR, ddPCR, and mNGS Stratified by Gender

| **method** | **gender** | **TP** | **FP** | **TN** | **FN** | **Sensitivity (%)** | **Specificity (%)** | **PPV (%)** | **NPV (%)** |
| --- | --- | --- | --- | --- | --- | --- | --- | --- | --- |
| MDO-dPCR | male | 24 | 9 | 23 | 20 | 54.55 | 71.89 | 72.72 | 53.49 |
|  | female | 6 | 2 | 6 | 7 | 46.15 | 75.00 | 75.00 | 46.15 |
| ddPCR | male | 12 | 3 | 8 | 14 | 46.15 | 72.73 | 80.00 | 36.36 |
|  | female | 4 | 1 | 3 | 3 | 57.14 | 75.00 | 80.00 | 50.00 |
| mNGS | male | 19 | 2 | 1 | 2 | 90.48 | 33.33 | 90.48 | 33.33 |
|  | female | 8 | 1 | 0 | 1 | 88.89 | 0.00 | 88.89 | 0.00 |

Table S4. Diagnostic Performance of MDO-dPCR, ddPCR, and mNGS Stratified by Age Group

| **method** | **Age (years)** | **TP** | **FP** | **TN** | **FN** | **Sensitivity (%)** | **Specificity (%)** | **PPV (%)** | **NPV (%)** |
| --- | --- | --- | --- | --- | --- | --- | --- | --- | --- |
| MDO-dPCR | < 64 | 7 | 8 | 15 | 11 | 38.89 | 65.22 | 46.67 | 57.70 |
|  | ≥ 64 | 23 | 3 | 14 | 16 | 58.97 | 82.35 | 88.46 | 46.67 |
| ddPCR | < 64 | 8 | 1 | 5 | 13 | 38.10 | 83.33 | 89.89 | 27.78 |
|  | ≥ 64 | 8 | 3 | 6 | 4 | 66.66 | 66.66 | 72.72 | 60.00 |
| mNGS | < 64 | 10 | 2 | 2 | 1 | 90.91 | 50.00 | 83.33 | 66.67 |
|  | ≥ 64 | 18 | 1 | 1 | 0 | 100.00 | 50.00 | 94.74 | 100.00 |
